# Dynamic One‐Dimensional Beam Shaping via a Mechanically Tunable Bilayer Metasurface

**DOI:** 10.1002/nap2.70093

**Published:** 2026-04-20

**Authors:** Jun Seok Yoon, Sangtae Jeon, Yu Geun Ki, Beom Jin Jin, Hyeongseop Lim, Soo Jin Kim

**Affiliations:** ^1^ School of Electrical Engineering Korea University Seoul Korea; ^2^ Department of Semiconductor Systems Engineering Korea University Seoul Korea

**Keywords:** accelerating beam, Airy beam, cosine‐Gauss beam, tunable metasurfaces, varifocal lens

## Abstract

Tunable metasurfaces have emerged as a promising platform for active wavefront control beyond the static functionality of conventional designs. Although mechanical actuation in bilayer metasurfaces offers large tuning ranges and robust operation, previous studies have predominantly focused on two‐dimensional (2D) beam control that requires complex phase profiles and is subject to design constraints arising from the limited degrees of freedom available in two‐axis modulation. In contrast, one‐dimensional (1D) optical beams—widely used in line‐scan imaging and light‐sheet microscopy—remain largely unexplored for mechanical tuning, despite their potential for simpler implementation. In this work, we propose a simple yet effective design principle for a bilayer metasurface capable of actively tuning various 1D optical beams. The approach exploits the additional design freedom along the axis orthogonal to the phase profiles used for 1D beam shaping, enabling controllable beam modulation with minimal structural complexity. We validate the feasibility of the proposed principle through theoretical analysis and experimental demonstration at a near‐infrared wavelength.

## Graphical abstract

We propose a generalized method for designing a bilayer metasurface for mechanically tuning one‐dimensional beams. The lateral displacement between layers directly affects the final phase profile of the system, thereby modulating the beam characteristics. Orthogonal decoupling of the modulation and wavefront shaping enables simple and versatile 1D beam tuning.

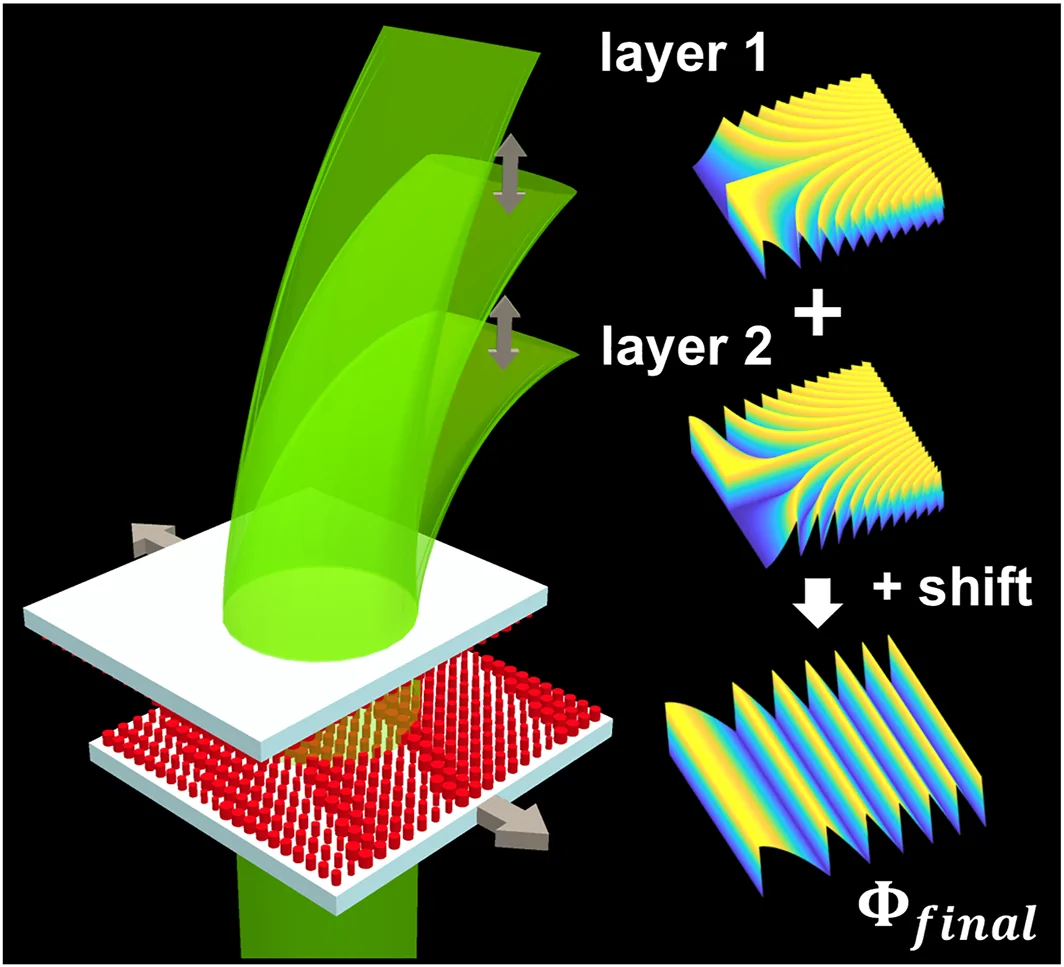

## 1 Introduction

Optical metasurfaces are two‐dimensional (2D) arrays of nanostructures arranged on a subwavelength scale. Light–matter interactions between incident light and these nanostructures enable control over optical properties such as amplitude, phase, and polarization. These capabilities enable metasurfaces to provide precise wavefront control with a compact form factor that can replace conventional bulky optics. Previous studies on wavefront shaping using metasurfaces have led to rapid advances in various applications, including lenses [1, 2, 3], beam deflectors [4, 5], and holography [6, 7]. Despite these advancements, the limited functionality of fixed metasurface designs has recently driven growing interest in tunable metasurfaces with multifunctional operations. Unlike passive multifunctional metasurfaces that generate multiple outputs by interleaving segments designed for different outputs or by optimizing a single pattern to support multiple functionalities [8, 9, 10], actively tunable metasurfaces require external stimuli to modify either the optical properties of the materials or the structural configuration of the system. Some earlier studies applied electrical bias to conductive materials or phase‐change materials for active tuning by changing material properties [11, 12, 13]. Other studies applied stress to flexible and stretchable substrates [14, 15, 16] or employed mechanical actuation in bilayer systems to reconfigure the overall structure [17, 18, 19, 20]. Among the various tuning mechanisms, mechanical tuning based on bilayer metasurfaces has been extensively studied because of its wide tuning range and rapid response [21, 22, 23, 24, 25]. Such mechanical tuning is also compatible with micro‐electromechanical systems (MEMS) that use electrical bias to enable precise mechanical displacement within the device [26].

These recent advances in tunable metasurfaces, however, have mostly focused on manipulating 2D optical wavefronts. Although 2D beams are essential for many optical systems, one‐dimensional (1D) beams also play indispensable roles in a wide range of applications. In many practical architectures, line‐ or sheet‐like illumination is intentionally used to confine light tightly along one axis while maintaining extended coverage along the orthogonal axis. This geometry confines illumination near the intended line or plane, thereby reducing unnecessary exposure outside the region of interest. In addition, illumination and detection can be performed line by line or plane by plane during each measurement. As a result, these 1D‐beam‐based schemes are distinct from point‐focused 2D approaches that rely on point‐by‐point scanning and may incur additional exposure and acquisition overhead. For example, light‐sheet microscopy enables high‐contrast volumetric fluorescence imaging by illuminating a thin optical plane, thereby enabling rapid acquisition with minimal photodamage in biological studies [27, 28, 29, 30, 31]. Similarly, line‐scan imaging sequentially scans a 2D plane using a 1D beam, achieving high throughput and efficiency in bioimaging and hyperspectral imaging [32, 33, 34, 35]. Despite their broad utility, tunable metasurfaces for controlling 1D beams remain virtually unexplored. Moreover, most mechanically reconfigurable metasurfaces have been developed primarily for 2D wavefront tuning, and their design procedures are often tailored to specific target functionalities. As a result, directly translating these approaches to 1D beam control does not necessarily lead to a unified principle that can be applied across diverse 1D phase functions with a single actuation mechanism. In contrast, we introduce a conceptually simple yet versatile design strategy based on a generalized design principle for tunable 1D wavefront shaping. Under this principle, the same bilayer architecture can modulate different beams through a single in‐plane translation, thereby tuning the effective coefficient of the target phase function encoded in the bilayer. Specifically, a controlled in‐plane translation between two metasurface layers—along the axis orthogonal to the intrinsic phase profiles used for 1D wavefront shaping—continuously alters their combined phase distribution, enabling active modulation of the transmitted 1D beam in a highly predictable manner. This approach simplifies the phase design process while maintaining dynamic tunability, providing a new route toward mechanically reconfigurable metasurfaces. Using both analytical modeling and experimental verification, we demonstrate effective 1D beam modulation in a bilayer metasurface architecture, validating the proposed design principle.

## 2 Results and Discussion

A schematic of the proposed design is shown in Figure 1a. It is composed of two parallel metasurface layers separated by a small vertical gap. The metasurfaces consist of an array of polysilicon nanocylinders on a quartz substrate, ensuring polarization independence owing to their symmetric structure (Figure 1b). The lateral actuation along the *y*‐axis from the centrally aligned state introduces a displacement *d* between the two layers. This additional degree of freedom associated with *y*‐directed displacement in 1D wavefront shaping enables continuous tuning of the output light beam. The design procedure for the phase profiles of both layers is shown in Figure 1c,d. To tune a 1D beam generated by the phase function f(x), the phase profiles of the two metasurface layers can be expressed as

(1) $$\varphi_{\text{layer}1}\left(x,y\right)=-\alpha yf\left(x\right),$$

(2) $$\varphi_{\text{layer}2}\left(x,y\right)=\alpha yf\left(x\right),$$

where (*x*, *y*) denotes the coordinates of the metasurfaces and *α* is a coefficient with a value of 1 μm^−1^. Without any actuation, the wavefront of the normally incident light remains unchanged after passing through both layers, as the sum of Equations (1) and (2) is zero. However, after translating the two layers by ± *d*/2 along the *y*‐axis, the phase profile of the system illustrated in Figure 1e can be written as

(3) $$\begin{array}{cc} \varphi_{\text{final}} & =\varphi_{\text{layer}1}\left(x,y-d/2\right)+\varphi_{\text{layer}2}\left(x,y+d/2\right)\\ & =\alpha df\left(x\right) \end{array}.$$

**FIGURE 1:**
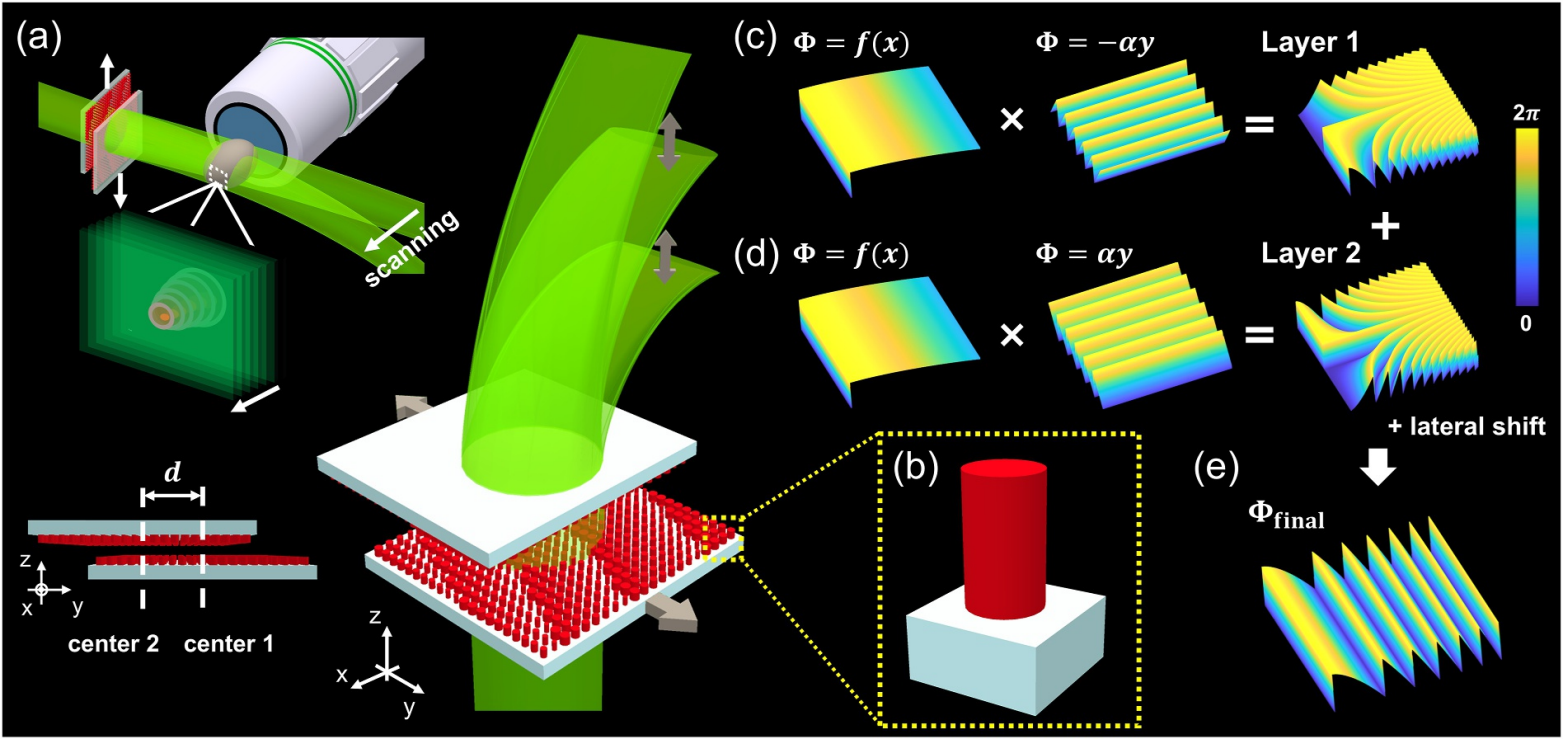
Proposed mechanically tunable system for active 1D wavefront shaping and the design principle of the metasurface phase profile. (a) Schematic of a potential application in volumetric light‐sheet microscopy and the operation of the overall system. (b) Unit cell consisting of an array of Si nanocylinders with a height of 390 nm, a period of 370 nm, and a radius ranging from 70 to 150 nm on a quartz substrate. (c–e) Design principle of the phase profile of the system. (c, d) The phase profile of each layer corresponds to the target function f(x) multiplied by –*αy* and +*αy*, respectively. (e) The final phase profile corresponds to the target function multiplied by the displacement term.

The opposite signs of Equations (1) and (2) cancel out all terms related to *y*, leaving only *x*‐ and *d*‐dependent terms. The final phase profile is therefore given by the product of the target phase function f(x) and the total displacement *d*. The relationship between the displacement and the modulated beam characteristics depends on the phase function and the specific beam property being considered, exhibiting either linear or nonlinear behavior. From Equations ((1), (2), (3)), a wide range of 1D light beams described by the function f(x) can be modulated using the proposed system, as demonstrated in the following sections.

To verify the tunability of the proposed system, numerical simulations were performed using the finite‐difference time‐domain (FDTD) method. The phase functions and parameters for each light beam are presented in Table 1. The parameters were chosen in accordance with the size of the metasurface layers, and the incident wavelength *λ*_0_ is fixed at 950 nm. To express the final phase profile shown in Equation (3), an interlayer spacing smaller than the Talbot distance of $z_{T}=2p^{2}/\lambda$ is required to suppress diffraction that would otherwise distort the wavefront incident on layer 2 [25]. Throughout the simulations, the interlayer spacing was set to 200 nm, which is smaller than the Talbot distance. A detailed analysis of the interlayer spacing is provided in Section 5 in Supporting Information S1. Since the optical profiles are formed on the quartz substrate, the effective wavelength and optical properties were calculated using its refractive index.

**TABLE 1:** Phase functions and parameters used for the design.

	f(x)	Parameters
Cosine‐Gauss beam	$-\frac{2\pi n_{0}}{\lambda_{0}}\left|x\right|$	*n*_0_ = 1/60
Lens	$-\frac{\pi }{\lambda_{0}f_{0}}x^{2}$	*f*_0_ = 800 μm
Airy beam	$\frac{1}{3}{\left(-\frac{2\pi x_{0}}{\lambda_{0}f_{0}}\right)}^{3}x^{3}$	*f*_0_ = 50 μm *x*_0_ = (0.1)^1/3^ μm
Logarithmic accelerating beam	2πe−1a2bλ01−e−xa	*a* = 10 μm *b* = 0.01 μm^−1^

Considering practical applications of 1D beams, four representative beams—two focusing beams and two accelerating beams—were designed and numerically analyzed. These two categories were chosen because their optical properties are highly relevant to practical 1D beam applications. The first two beams, the cosine‐Gauss beam (CGB) and the varifocal lens, exhibit focusing behavior and are tuned by varying the subtended angle (2*θ*) and the numerical aperture (NA), respectively. The ideal cosine beam is an exact solution of the free‐space Helmholtz equation with nondiffracting properties and can be regarded as a one‐dimensional counterpart of the Bessel beam, formed by the interference of two symmetrically tilted waves at ± *θ* [36, 37]. The Gaussian apodization of the cosine beam yields a CGB with a finite diffraction‐resistant propagation range. Similar to the zero‐order Bessel beam generated by a circular axicon, the cosine beam can be generated with a Fresnel biprism. The phase profile of the cosine beam is therefore given by $\varphi \left(x\right)=-k_{0}n_{0}\left|x\right|$. Here, *k*_0_ is the wave vector in vacuum, and *n*_0_ is equal to nsinθ, where *n* is the refractive index of the propagating medium and *θ* is the half angle between the two symmetrically tilted wave components. Figure 2a shows the normalized intensity profiles of the simulated CGBs with different subtended angles, corresponding to displacements *d* ranging from 6 to 12 μm. The waist radius *ω*_0_ of the incident Gaussian beam was set to 10 μm throughout the simulations. In the analytical derivation in Section 3 in Supporting Information S1, the full width at half maximum (FWHM) and the distance *z*_max_ over which the CGB is considered nondiffracting are given by $\text{FWHM}=\frac{0.252\lambda_{0}}{\alpha n_{0}d}$ and $z_{\max}=\frac{n\omega_{0}}{\alpha n_{0}d}$. The theoretical and simulated values of FWHM and *z*_max_ are plotted in Figure 2b,c as a dashed black line and red circles, respectively. The simulated values are inversely proportional to the displacement, in agreement with theoretical predictions.

**FIGURE 2:**
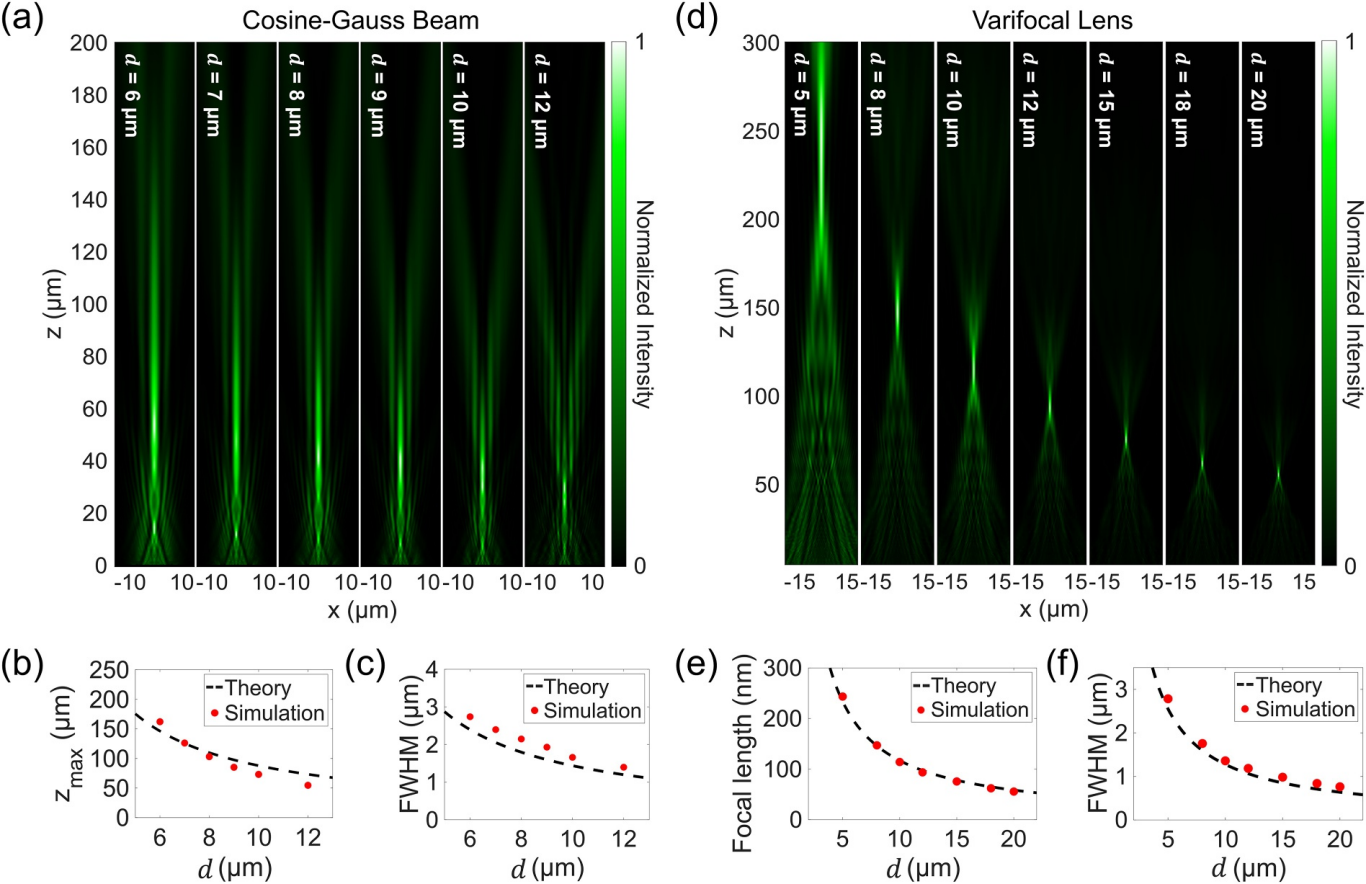
Simulated analysis of the cosine‐Gauss beam and the varifocal lens with varying the subtended angle and NA. (a) Normalized field intensity distributions for cosine‐Gauss beams with different displacements. (b) Theoretical (dashed black line) and simulated (red circles) *z*_max_ of the cosine‐Gauss beams. (c) Theoretical (dashed black line) and simulated (red circles) FWHM of the cosine‐Gauss beams. (d) Normalized field intensity distributions for the varifocal lens with different displacements. (e) Theoretical (dashed black line) and simulated (red circles) focal length of the varifocal lens. (f) Theoretical (dashed black line) and simulated (red circles) FWHM of the varifocal lens.

To further illustrate the NA‐based tuning, the varifocal lens, one of the most widely studied active optical elements, is considered another example. Here, the phase profile of the lens is expressed using the paraxial approximation. Figure 2d shows the optical profiles of the lens for *d* varying from 5 to 20 μm. Using Equation (3), the focal length of the lens can be expressed as $f=n\cdot f_{0}/\alpha d$ where *n* is the refractive index of the propagating medium. The simulated (red circles) and theoretical (dashed black line) focal lengths are plotted in Figure 2e. The simulated results vary inversely with the displacement and are in close agreement with the theoretical curve. Figure 2f shows the FWHM at each focal point. The dashed black line indicates the theoretical values calculated using $\text{FWHM}=0.8858\lambda f/W_{x}$, where *W*_*x*_ is the width of the lens along the *x*‐axis (see Section 4 in Supporting Information S1). The red circles represent simulation results and are consistent with the theoretical curve. These results demonstrate the effectiveness of NA‐based tuning for controlling the focusing behavior of the proposed metasurface system.

To extend the design concept, another class of beams is considered, namely accelerating beams with curved propagation trajectories and nondiffracting behavior. Owing to these characteristics, accelerating beams are often employed in light‐sheet microscopy to achieve a larger field of view and higher imaging rates [29, 30]. One well‐known example is the Airy beam, which is characterized by its parabolic trajectory. The most common method of generating an Airy beam is to take the Fourier transform of a cubic phase [38, 39, 40, 41, 42]. Instead of placing an additional lens after the metasurfaces, the phase profile of the lens, $-\frac{\pi }{\lambda_{0}f_{0}}x^{2}$, is added to layer 2 to perform the optical Fourier transform. The lens phase term is independent of *d* to keep the starting point of the Airy beam constant. The normalized optical profiles of Airy beams with a displacement from −15 to 15 μm are presented in Figure 3a. The horizontal white dashed line shows the Fourier plane of the lens term on layer 2. As the Airy beam is generated at the Fourier plane, the coordinate origin (*z* = 0) is defined at the starting point of the beam. The dashed white curve starting from the Fourier plane represents the calculated trajectory of the Airy beam. The trajectory is calculated from the total deflection of the beam along the propagation, which is given by [40]

(4) $$\Delta =\frac{{fz}^{2}}{{4k^{2}x}_{0}^{3}\left(f+z\right)\alpha d},$$

where k=2π/λ, *f* is the focal length of the lens, and *x*_0_ is a scale factor in Table 1. According to Equation (4), a smaller displacement results in a larger deflection as the beam propagates. The intensity cross‐sections along the *z*‐axis when *d* = −10 μm are plotted in Figure 3b. The first main lobe of the beam is deflected toward the −*x* direction. Although the intensity of the main lobe decreases as the beam propagates, the shape of the lobe is largely preserved, indicating that the accelerating behavior of the Airy beam is maintained under mechanical tuning.

**FIGURE 3:**
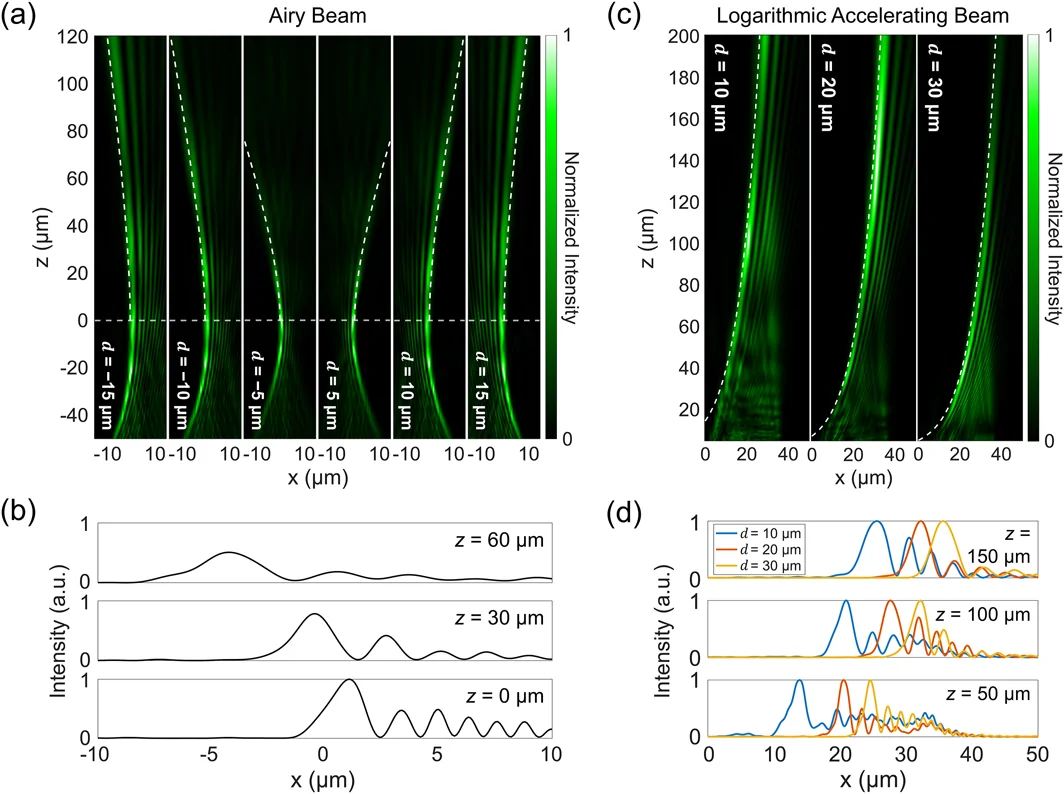
Simulated analysis of accelerating beam tuning with different trajectories. (a) Normalized field intensity distributions for the Airy beam with a parabolic trajectory under different displacements. (b) Intensity cross‐sections at *z* = 0, 30, and 60 μm when *d* = −10 μm. (c) Normalized field intensity distributions for the accelerating beam with a logarithmic trajectory under different displacements. (d) Overlaid intensity cross‐sections of the three curves at 50, 100, and 150 μm. Each curve is normalized to its own maximum value.

In addition to the Airy beam, accelerating beams can be realized by shaping their propagation trajectories through caustic design [43, 44, 45, 46]. Unlike the Fourier‐transform‐based approach, the caustic design method generates the accelerating trajectory directly from the corresponding spatial phase functions. Since the caustic is defined as an envelope of tangential slopes, each point *x* on the metasurface plane is related to a point on the curve satisfying $c^{\prime }\left(z\right)=\frac{dc\left(z\right)}{dz}=\frac{c\left(z\right)-x}{z}$, where *c*(*z*) is defined as the arbitrary trajectory curve. Therefore, the phase distribution on the metasurface can be calculated from

(5) $$\frac{d\varphi \left(x\right)}{dx}=\frac{{kc}^{\prime }\left(z\right)}{\sqrt{1+{\left[c^{\prime }\left(z\right)\right]}^{2}}},$$

where *k* is the wavenumber [44, 45, 46]. Among the various types of curves, the natural logarithmic curve x=aln(bz) is chosen as an example of a caustic trajectory. Following this approach, the phase profile $f\left(x\right)=\frac{{2\pi e}^{-1}a^{2}b}{\lambda_{0}}\left(1-e^{-x/a}\right)$ is used, where *a* and *b* are arbitrary constants that determine the beam trajectory. As the constant *a* in f(x) also appears in the exponent, the displacement *d* affects the constant *b*, resulting in the trajectory x=aln(αdbz). The normalized intensity profiles of the accelerating beam with a logarithmic trajectory are shown in Figure 3c for displacements of 10, 20, and 30 μm. The white dashed line shows the theoretical target curve. As the trajectory is a function of the natural logarithm, the beam tuning can be described using logarithmic identities. Here, the curve for each displacement can be written as x=aln(10bz), x=aln(20bz), and x=aln(30bz). Using the product rule for logarithms, the trajectory when *d* = 20 and 30 μm can be rewritten as x=aln(10bz)+aln(2) and x=aln(10bz)+aln(3). This corresponds to a translation of x=aln(10bz) along the *x*‐axis. The translation is illustrated in Figure 3d using intensity cross‐sections at *z* = 50, 100, and 150 μm. The blue and red curves exhibit an average peak‐to‐peak distance of 6.67 μm, which is comparable to the theoretical value (6.93 μm) obtained from 10ln(2). Additionally, the blue and yellow curves indicate an average peak‐to‐peak distance of 10.67 μm over the three plots, in good agreement with the theoretical value of 10.99 μm corresponding to 10ln(3).

To experimentally demonstrate the tunability of the proposed system, we fabricated samples corresponding to two representative cases among the simulated beams—one with a focusing property and the other with an accelerating property. Specifically, the varifocal lens and the Airy beam were selected as representative examples of focusing and accelerating beams, respectively. The detailed fabrication process is described in Figure S3 in Supporting Information S1. The same phase profiles were used in both simulations and experiments. Top‐view and tilted‐view scanning electron microscopy (SEM) images of the fabricated sample are shown in Figure 4a. The schematic of the experimental setup for measuring the output beam profile is illustrated in Figure 4b (see Methods). Due to the difficulty of aligning the two metasurface layers, the interlayer spacing was measured to be about 10 μm. Figure 4c shows the measured optical profile of the varifocal lens along the *z*‐axis at different displacements. The motorized stage was moved along the optical axis, recording beam profile images of the *xy* plane with a step size of 1 μm. The reconstructed stack of 2D *xy* plane images was used to obtain the 3D field profile along the propagation direction. The *y* = 0 slice of the 3D field profile corresponds to the *xz* plane of the optical profile for analysis of the beam properties. The theoretical and measured focal lengths are plotted in Figure 4d. The dashed black line represents the theoretical focal length calculated using the same equation as in Figure 2e. The experimental results shown by red circles demonstrate the tunability of the system and generally follow the overall trend of the theoretical curve. Deviations between experiments and theory are mainly attributed to the imperfect experimental setup and fabrication errors. The large sample size leads to a larger interlayer distance despite the nearly parallel alignment, resulting in a shorter focal length. Moreover, the fabrication errors that cause tapered sidewalls and an enlarged bottom radius also contribute to a reduced focal length. A detailed analysis of the discrepancies is presented in Sections 5–7 in Supporting Information S1. The normalized intensity cross‐section along the *x*‐axis at the focal point is plotted in Figure 4e. The measured FWHM values are plotted as red circles with error bars in Figure 4f. The theoretical curve is plotted as a dashed black line, calculated using the same equation as that used in Figure 2f. The overall trend of the measured data agrees well with the theoretical curve at low NA. However, at higher NA, reduced focusing efficiency causes a larger FWHM. Notably, the fabricated sample exhibits a much shorter focal length, as shown in Figure 4d. As a result, the effective NA of each measurement differs from the designed value, leading to greater degradation of the focal spot.

**FIGURE 4:**
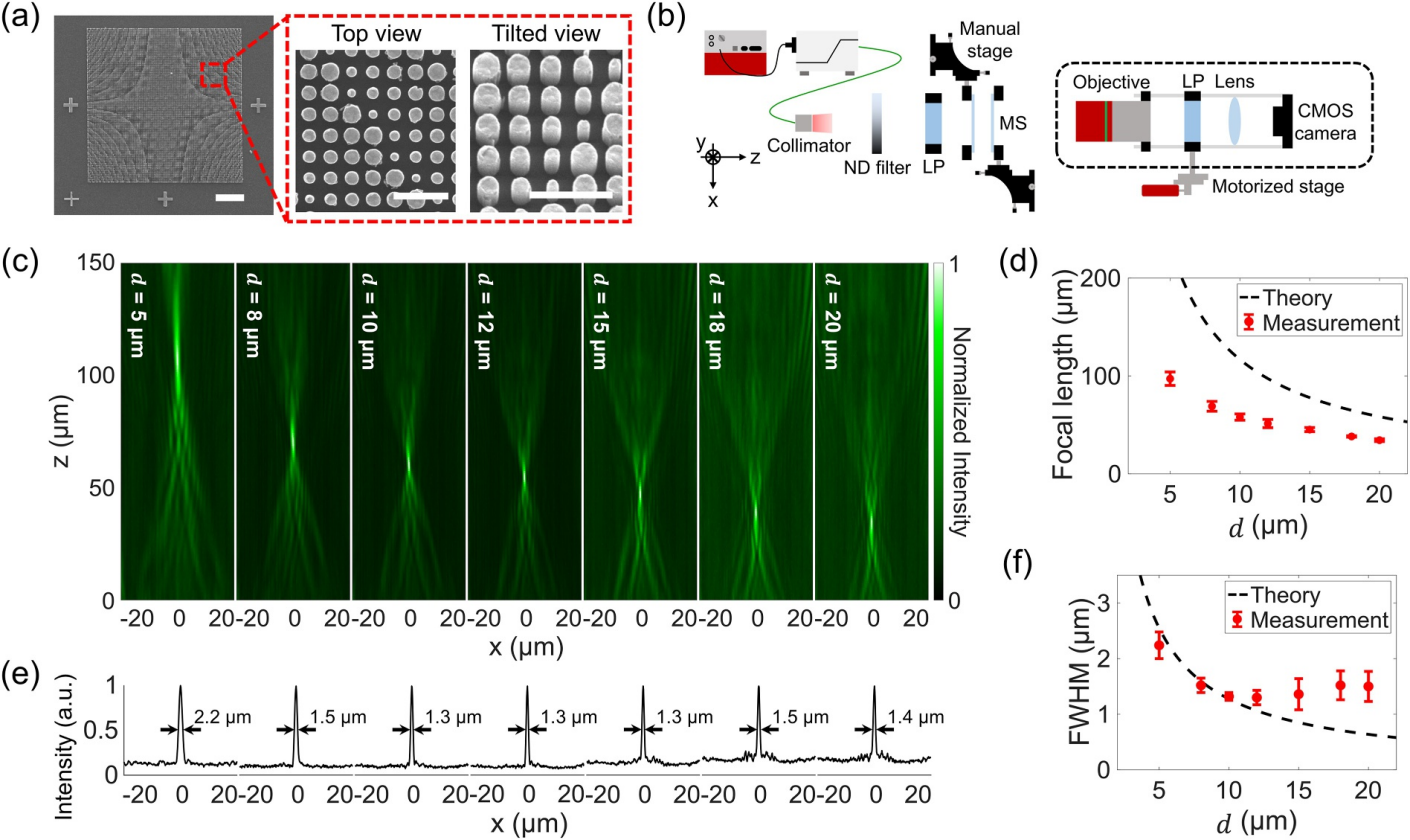
Experimental measurements of a varifocal lens. (a) SEM images of the fabricated metasurface. Scale bar: 10 μm. Magnified top‐view and tilted‐view SEM image (tilt angle 40°) of unit cells of the metasurface. Scale bar: 1 μm. (b) Schematic of the overall measurement setup. (c) Measured field images of the varifocal lens with different displacements. (d) Theoretical (dashed black line) and measured (red circles) focal lengths. The error bars are the standard deviation of focal lengths from repeated measurements. (e) Normalized intensity cross‐sections at each focal length. (f) Theoretical (dashed black line) and measured results (red circles) of the FWHM with error bars indicating the standard deviations from repeated measurements. LP = linear polarizer, MS = metasurfaces.

To further confirm the tunability of the system, we conducted an experimental demonstration of the Airy beam, as a representative example of an accelerating beam. The 1D Airy beam profiles for different displacements were measured using the same experimental approach as for the varifocal lens, which is illustrated in Figure 5a. Without actuation, the cubic phase term vanishes, leaving the lens term as shown in the central panel. The horizontal white dashed line shows the Fourier plane measured in the experiment. The focal length of the lens integrated on layer 2 was also shorter than the designed focal length, as observed in the varifocal lens sample. The beam trajectory shown by a white dashed curve is obtained from Equation (4) using the measured focal length. At the Fourier plane, the measured (red circles with error bars) and calculated (black dashed line) FWHM of the first lobe of the Airy beam are compared in Figure 5b,c. The intensity distribution after the Fourier transform is proportional to the square of the Airy function, ${\text{Ai}}^{2}\left(\frac{x}{x_{0}\cdot {\left(\alpha d\right)}^{1/3}}\right)$ where *x*_0_ is a scale factor in Table 1. The FWHM of the first lobe can therefore be approximated as $1.6\cdot x_{0}\cdot {\left(\alpha d\right)}^{1/3}$ [42]. Figure 5d shows the normalized intensity cross‐sections from *z* = 0 μm to *z* = 50 μm planes at 10 μm intervals when *d* = −8 μm, demonstrating the accelerating behavior of the Airy beam. In addition, Figure 5e–h show the positions of the maximum intensity of the Airy beam along the propagation axis when *d* = 8, 10, 12, and 15 μm, respectively. Similarly, Figure 5i–l show the positions of maximum intensity for *d* = −8, −10, −12, and −15 μm, respectively. The experimental results shown by colored circles are in close agreement with the theoretical curve calculated from Equation (4). The minor deviations and discontinuities observed at experimental points can be attributed to imperfections in the fabricated sample and light incident on single‐layer‐only regions and the unpatterned substrate (Section 7 in Supporting Information S1).

**FIGURE 5:**
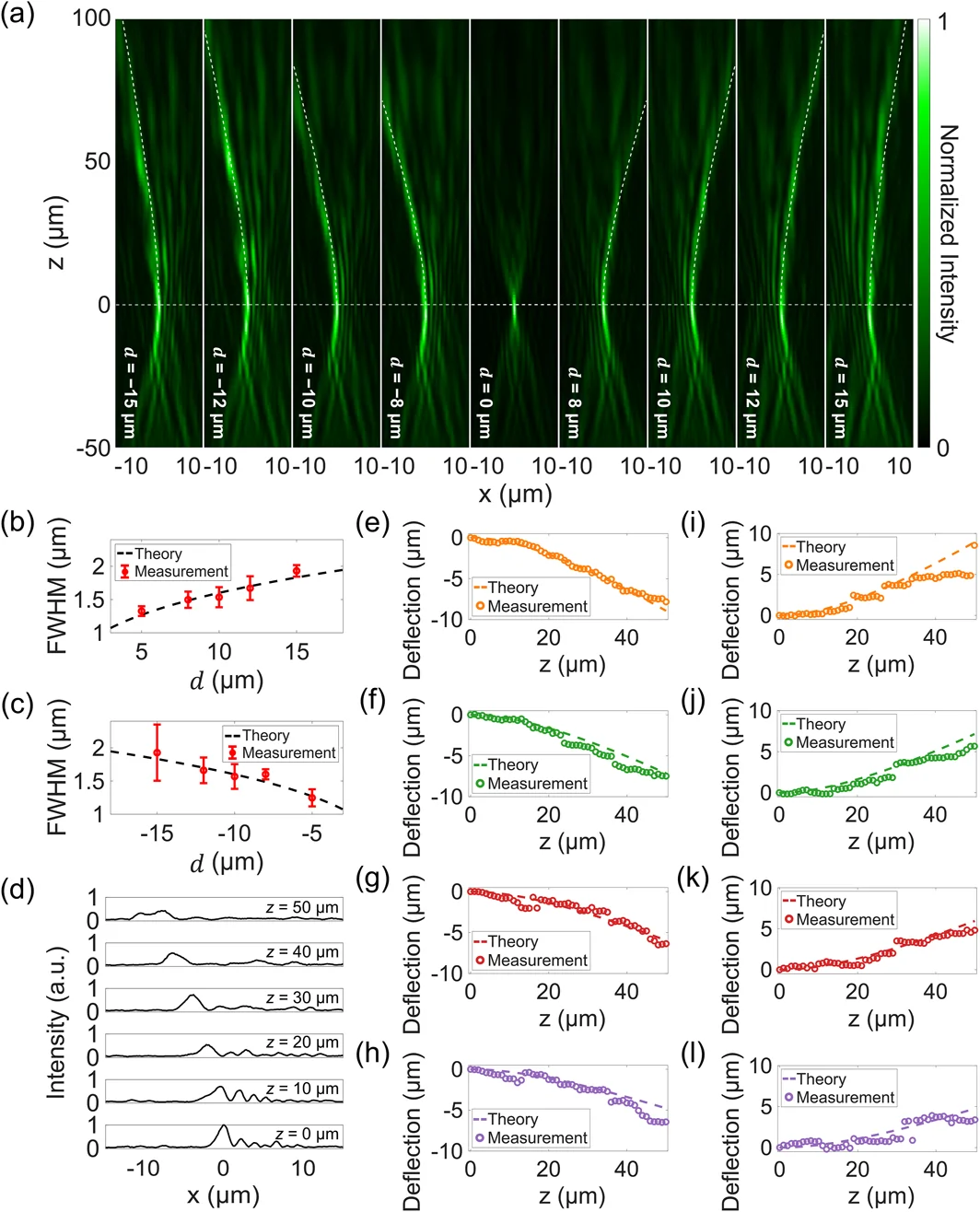
Optical characteristics of the tunable Airy beam. (a) Measured field images of the tunable Airy beam with different displacements. (b, c) Theoretical (dashed black line) and measured (red circles) FWHM of the main lobe of the Airy beam at the Fourier plane when (b) d>0 and (c) d<0. Error bars indicate the standard deviations from repeated measurements. (d) Normalized intensity cross‐sections at different *z* positions for *d* = −8 μm. (e–h) Theoretical (dashed line) and measured (colored circles) trajectory of the main lobe when *d* = 8, 10, 12, and 15 μm. (i–l) Theoretical (dashed line) and measured (colored circles) trajectory of the main lobe when *d* = −8, −10, −12, and −15 μm.

## 3 Conclusion

In conclusion, we have developed and experimentally demonstrated a bilayer metasurface system capable of mechanically tuning one‐dimensional (1D) optical beams based on a generalized design principle. Although most tunable metasurfaces have focused on two‐dimensional (2D) wavefront control, 1D beam manipulation remains largely unexplored despite the importance of 1D beams in microscopy and imaging applications, where they enable fast scanning and low phototoxicity. Our design uses orthogonal coordinates to introduce a controllable lateral displacement between the two layers, enabling dynamic modulation of the transmitted beam. The relationship between the displacement and the resulting beam properties is summarized in Table 2. Numerical simulations and experimental measurements confirm the relationship between the mechanical displacement and the resulting optical response. This design principle can be extended across spectral ranges by adjusting unit‐cell geometry and material properties, and the tuning range and sensitivity can be directly tailored through the phase profile coefficients. The lateral actuation mechanism is well suited to MEMS‐based implementations, offering low power consumption, fast response, and compact packaging. Overall, this work establishes a practical framework for mechanically tunable metasurfaces, providing adaptive wavefront shaping capability for imaging, sensing, and optical communication applications.

**TABLE 2:** The relation between displacement and optical properties.

	Optical characteristic	Relation
Cosine‐Gauss beam	*z*_max_ FWHM	$n\omega_{0}/\alpha n_{0}d$ $0.252\lambda_{0}/\alpha n_{0}d$
Lens	Focal length FWHM	$nf_{0}/\alpha d$ $0.8858\alpha \lambda_{0}f_{0}d/W_{x}$
Airy beam	Deflection (Δ) FWHM	$fz^{2}/4k^{2}x_{0}^{3}\left(f+z\right)\alpha d$ $1.6x_{0}{\left(\alpha d\right)}^{1/3}$
Logarithmic accelerating beam	Trajectory	x=aln(αdbz)

## 4 Methods

### 4.1 Numerical Simulations

To evaluate the feasibility of the system, numerical simulations were performed using the FDTD method. The cylindrical silicon nanocylinders (*n* = 3.59 + 0.001*i*) were placed on a quartz substrate (*n* = 1.45) to form a square array. Perfectly matched layer (PML) boundary conditions were used in all directions. The simulation domain is confined to the overlapping areas of the two layers, excluding single‐layered regions resulting from lateral displacement. The *x*‐polarized source was used throughout the simulations.

### 4.2 Experimental Setup

The supercontinuum laser (NKT Photonics, SuperK EVO) was used as a light source with a laser line tunable filter (Photon etc., LLTF Contrast). The fiber collimator (Thorlabs, F220FC‐532) was connected at the end of the fiber to collimate the incident light. The intensity of the incident light was controlled using the ND filter (Thorlabs, NDC‐50S‐3M). The linear polarizer (Thorlabs, LPNIRB100) was placed in front of the metasurface samples. The manual stages with differential adjusters (Thorlabs, MT3A/M) were used for micro‐scale lateral actuation. For precise alignment between the metasurface layers, a manual rotation stage (Thorlabs, RP005/M) was added to a stage for samples with a size of 3 cm by 3 cm loaded on the rotational cage mount (Thorlabs, CRM1T/M). The output beams were measured and magnified by using the objective lens (Mitutoyo, M Plan Apo NIR 20×) and a lens (*f* = 200 mm). The images were taken by a CMOS camera (Thorlabs, CS165MU/M). Another linear polarizer was added between a tube lens and a CMOS camera. The objective, a tube lens, a linear polarizer, and a CMOS camera were assembled in a 30 mm cage system and loaded on the motorized stage (Thorlabs, LNR25ZFS/M).

## Author Contributions

**Jun Seok Yoon:** conceptualization, methodology, data curation, formal analysis, validation, investigation, visualization, writing – original draft, writing – review and editing. **Sangtae Jeon:** conceptualization, methodology, investigation, formal analysis, data curation, validation. **Yu Geun Ki:** conceptualization, methodology, formal analysis, investigation. **Beom Jin Jin:** methodology, formal analysis. **Hyeongseop Lim:** methodology, formal analysis. **Soo Jin Kim:** conceptualization, methodology, investigation, supervision, funding acquisition, project administration, resources, writing – review and editing, writing – original draft. All authors have accepted responsibility for the entire content of this manuscript and approved its submission.

## Conflicts of Interest

The authors declare no conflicts of interest.

## Supporting information


Supporting Information S1

## Data Availability

The datasets generated and analyzed during the current study are available from the corresponding author upon reasonable request.
