# Supplementary material for: Dynamic One‐Dimensional Beam Shaping via a Mechanically Tunable Bilayer Metasurface
Source: Nanophotonics. 2026 Apr 20;15(8):e70093. doi: 10.1002/nap2.70093 (PMC13642623; doi:10.1002/nap2.70093)
Supplement: Supplementary file 1 [file NAP2-15-e70093-s001.pdf]

Supporting Information

Dynamic One-Dimensional Beam Shaping via a Mechanically Tunable Bilayer Metasurface

*Jun Seok Yoon, Sangtae Jeon, Yu Geun Ki, Beom Jin Jin, Hyeongseop Lim and Soo Jin Kim**

1. Analysis on the lateral misalignment between metasurface layers

To minimize alignment errors, we perform the measurement with precise alignment using the linear and rotational stages in the measurement setup. Alignment marks were fabricated next to the metasurfaces to track the displacement. To estimate undesirable alignment error that might occur during the experiment, we investigate the behavior of optical beam for various misalignment conditions by theoretical analysis and experimental measurements. Since the misalignment along the *y*-axis (δ_y) does not influence the beam generation and only affects *d*, the misalignment along the *x*-axis (δ_x) is discussed in this section. When δ_x is included in the phase function, the additional terms unrelated to the initial beams are added, leading to distortion on the output beam. For example, for the total phase profile of the varifocal lens, δ_x modifies the equation to

$$\begin{aligned}\varphi_{total} &= -\left(y-\frac{d}{2}\right)\left[-\frac{\pi}{\lambda f}(x+\delta_x)^2\right]+\left(y+\frac{d}{2}\right)\left(-\frac{\pi}{\lambda f}x^2\right)\\ &= \frac{\pi y}{\lambda f}(2\delta_x x+\delta_x^2)-\frac{\pi d}{\lambda f}\left(x+\frac{\delta_x}{2}\right)^2-\frac{\pi d\delta_x^2}{4\lambda f}\end{aligned} \tag{S1}$$

resulting in a function of both *x* and *y*, which no longer generates the 1D optical beam. The same approach can also be applied to other beams. For the experimental demonstration, the beam profile with misalignment at the $y = 0$ plane is measured for the calibration of alignment, as shown in Figure S1. The displacement d between layers is set as 8 μm. The experimental setup is illustrated in Figure S1a, where the misaligned value δ_x is applied at layer 1. In Figure S1b, the focal point of the lens moves in the $-x$ direction as δ_x increases, which shows good agreement with Equation S1. The distortion is also observed in Figure S1c. The cubic phase term introduces additional terms related to δ_x. The phase profile of a lens implemented only in layer 2 remains unaffected by the misalignment, maintaining the uniform beam position.

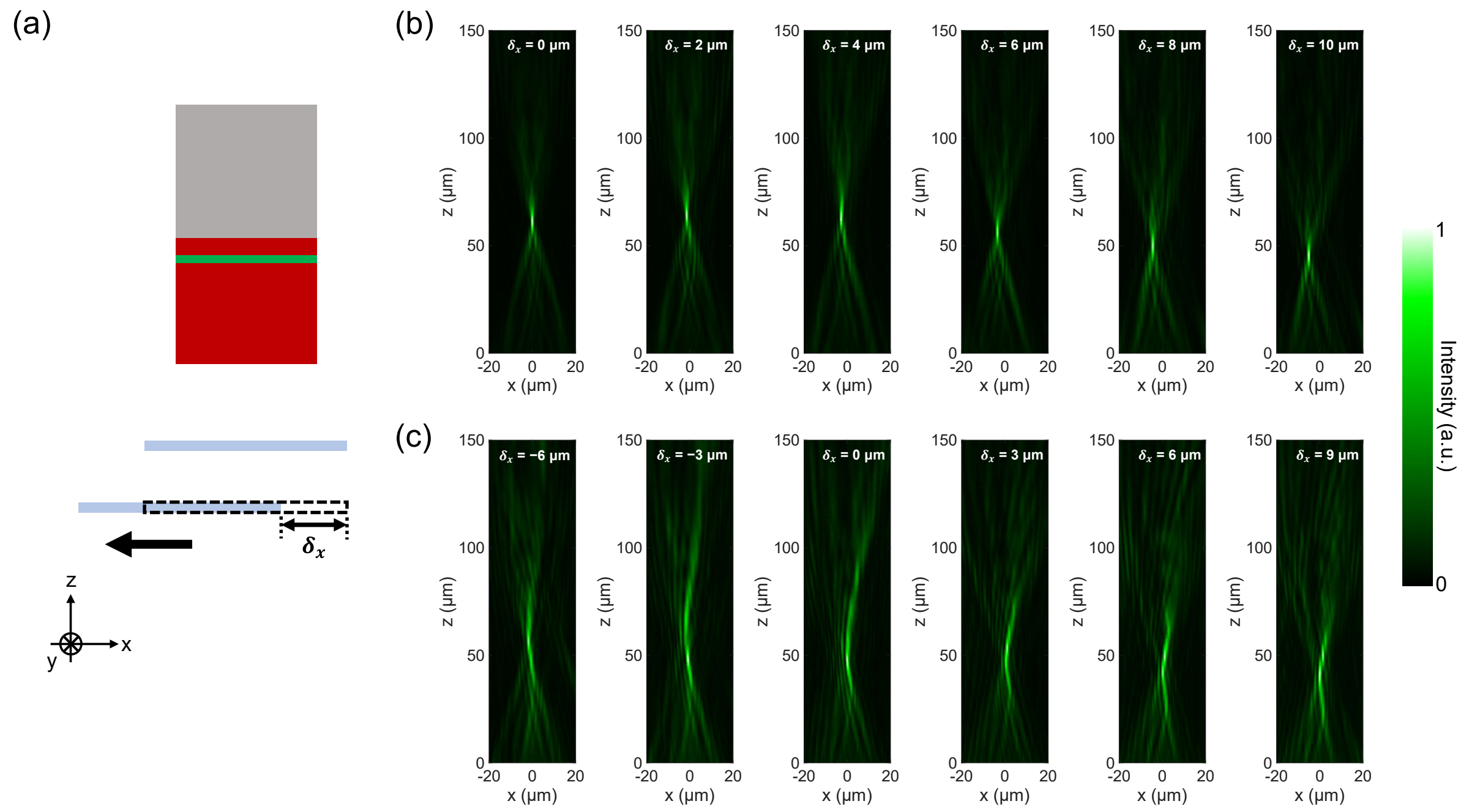

Figure S1. Experimental results for misalignment of the bilayer metasurfaces. (a) Schematic of the experimental setup for applying δ_x to layer 1. (b) Normalized optical profile of the varifocal lens depending on the value of δ_x. (c) Normalized optical profile of the Airy beam depending on the value of δ_x.

2. Unit cell design and sample fabrication process

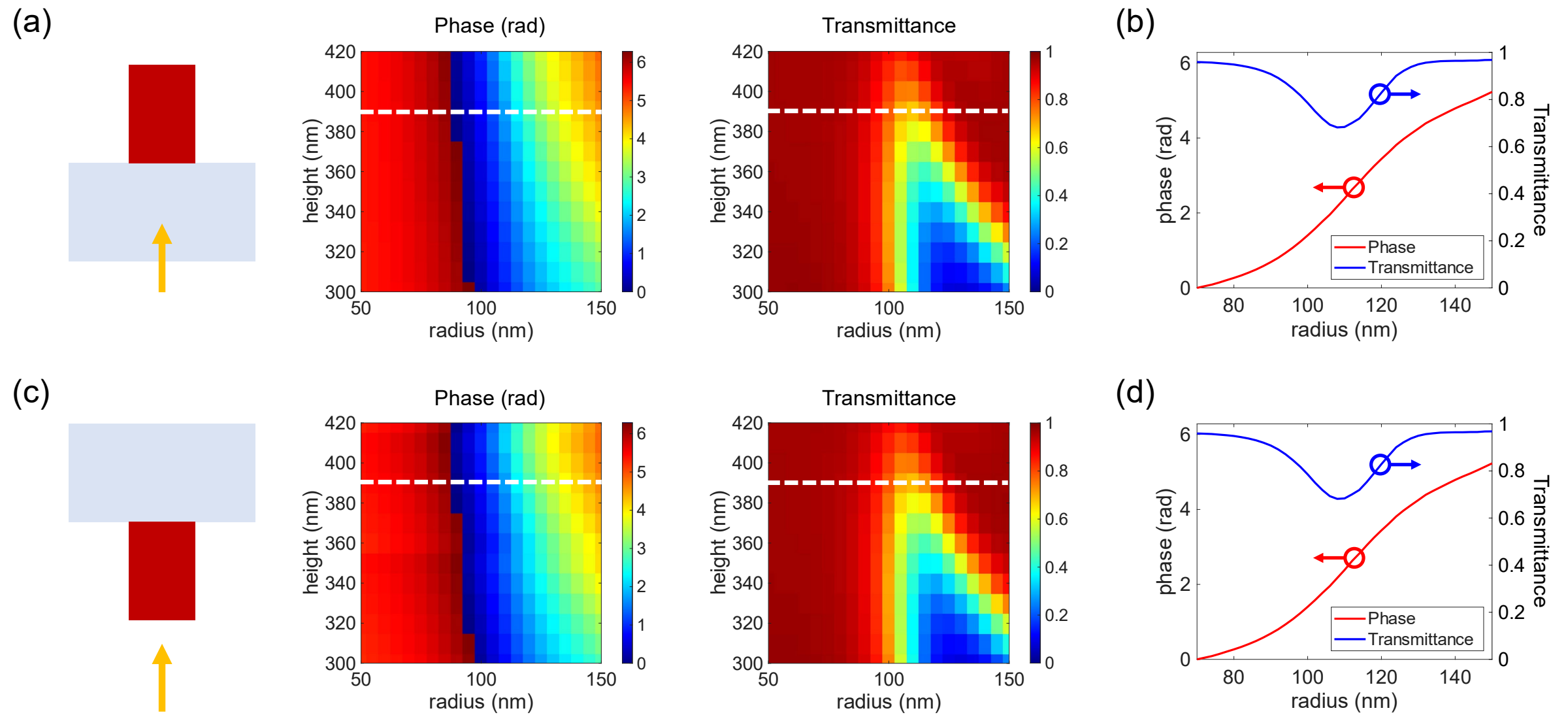

Figure S2. Simulated phase and transmittance of the unit cell. (a) Simulated phase and transmittance of the unit cells of layer 1 depending on the radius and height at the wavelength of 950 nm with a period of 370 nm. The white dashed line indicates the selected height of 390 nm. (b) Phase and transmittance of the final unit cell library of layer 1. (c) Simulated phase and transmittance of the unit cells of layer 2 depending on the radius and height. (d) Phase and transmittance of the final unit cell library of layer 2.

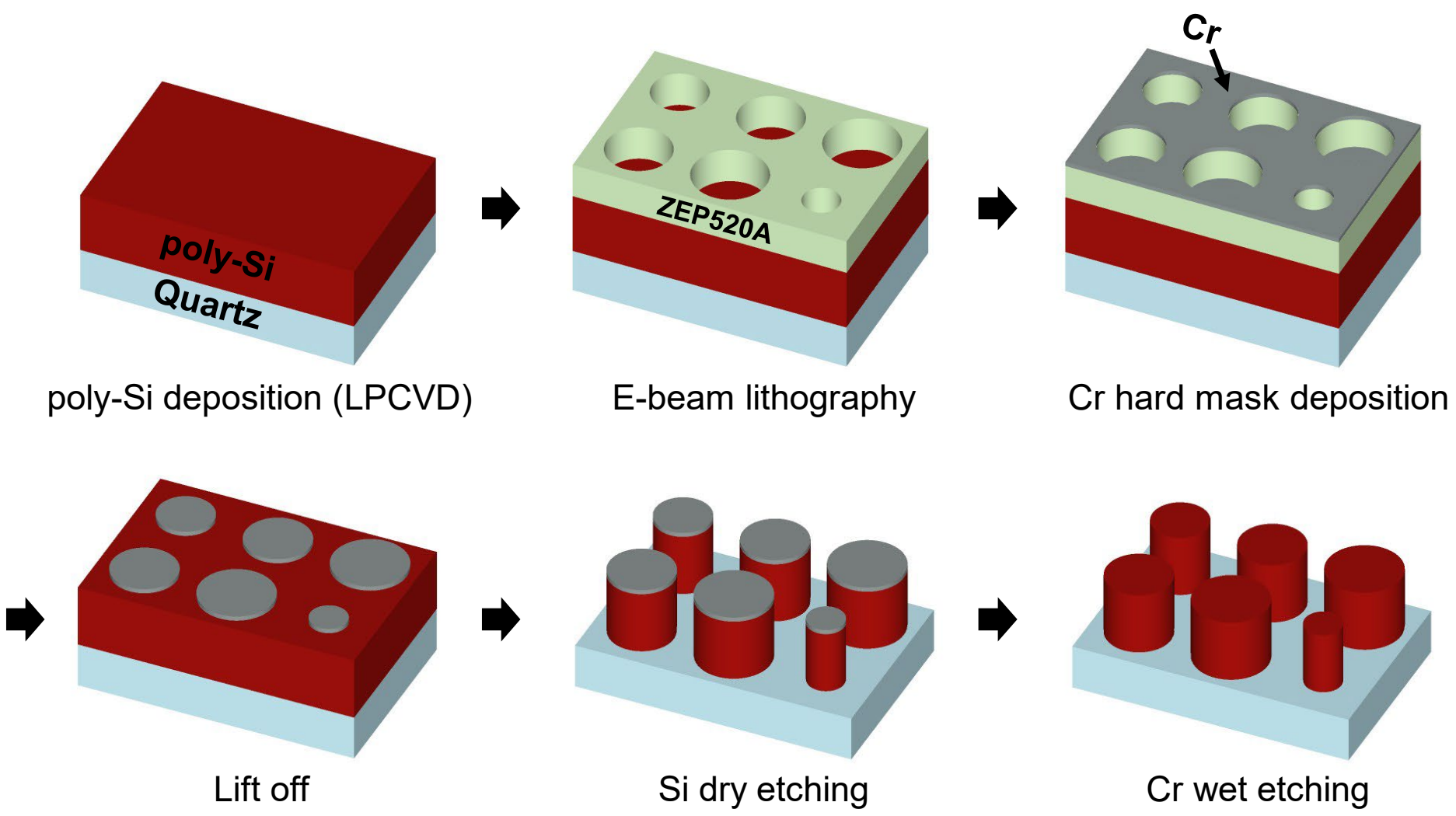

Figure S3. The fabrication process of the metasurface samples.

3. Analysis on cosine-Gauss beam

To analyze the optical properties of the cosine-Gauss beam (CGB), the electric field is calculated based on the Fresnel-Kirchhoff integral.[1] The electric field produced by the metasurfaces and the incident Gaussian beam at point (x', y', 0) is

$$E(x',y',0) = E_0 e^{-\frac{x'^2+y'^2}{\omega_0^2}} e^{-i\alpha d n_0 k_0 |x'|} \tag{S2}$$

where E_0 is the amplitude of the initial electric field, ω_0 is the radius of the beam waist of the incident Gaussian beam, k_0 is the incident wave vector, and $\alpha d n_0$ equals $n \sin\theta$ where n is the refractive index of the propagating medium and θ is half of the subtended angle. By substituting $\alpha d n_0 k_0$ into κ, the electric field at point (x, y, z) calculated by the Fresnel-Kirchhoff integral is given by

$$\begin{aligned} E(x,y,z) &= \frac{e^{ikz}}{i\lambda z} \iint E(x',y',0) e^{\frac{ik}{2z}(x-x')^2} e^{\frac{ik}{2z}(y-y')^2} dx'dy' \\ &= \frac{E_0 e^{ikz}}{i\lambda z} e^{\frac{ik}{2z}(x^2+y^2)} \cdot \int_{-\infty}^{\infty} e^{-\frac{x'^2}{\omega_0^2}} e^{-i\kappa|x'|} e^{\frac{ikx'^2}{2z}} e^{-\frac{ikxx'}{2z}} dx' \cdot \int_{-\infty}^{\infty} e^{-\frac{y'^2}{\omega_0^2}} e^{\frac{iky'^2}{2z}} e^{-\frac{ikyy'}{2z}} dy'. \end{aligned} \tag{S3}$$

Setting

$$\begin{gathered} \frac{E_0 e^{ikz}}{i\lambda z} e^{\frac{ik}{2z}(x^2+y^2)} = F \\ \int_{-\infty}^{\infty} e^{-\frac{x'^2}{\omega_0^2}} e^{-i\kappa|x'|} e^{\frac{ikx'^2}{2z}} e^{-\frac{ikxx'}{2z}} dx' = U_x(x,z) \\ \int_{-\infty}^{\infty} e^{-\frac{y'^2}{\omega_0^2}} e^{\frac{iky'^2}{2z}} e^{-\frac{ikyy'}{2z}} dy' = U_y(y,z) \end{gathered} \tag{S4}$$

leads Equation S3 into

$$E(x,y,z) = F \cdot U_x(x,z) \cdot U_y(y,z) \tag{S5}$$

Here, $U_y(y, z)$ is a standard Gaussian beam propagation along the y-axis, which does not affect the properties of CGB generated by the phase function of x. To calculate $U_x(x, z)$, we substitute $\frac{kx}{z}$ into γ and $\frac{1}{\omega_0^2} - \frac{ik}{2z}$ into $a(z)$. Therefore, $U_x(x, z)$ becomes

$$\begin{aligned} U_x(x,z) &= \int_{-\infty}^{\infty} e^{-au^2} e^{-i\kappa|x'|} e^{-i\gamma x'} dx' \\ &= \int_0^{\infty} e^{-au^2} e^{-i(\kappa-\gamma)u} du + \int_0^{\infty} e^{-au^2} e^{-i(\kappa+\gamma)u} du \end{aligned} \tag{S6}$$

$$= \frac{\sqrt{\pi}}{2\sqrt{a}} \left[\mathrm{erfc}\left(i \frac{\kappa - \gamma}{2\sqrt{a}} \right) e^{-\frac{(\kappa-\gamma)^2}{4a}} + \mathrm{erfc}\left(i \frac{\kappa + \gamma}{2\sqrt{a}} \right) e^{-\frac{(\kappa+\gamma)^2}{4a}} \right]$$

where $x' = -u$. Around the paraxial region where the electric field of $E(x' > 0, y', 0)$ and $E(x' < 0, y', 0)$ overlap, it can be estimated that $|\gamma| \ll |\kappa|$, which leads to

$$\mathrm{erfc}\left(i \frac{\kappa - \gamma}{2\sqrt{a}} \right) \approx \mathrm{erfc}\left(i \frac{\kappa + \gamma}{2\sqrt{a}} \right) \approx \mathrm{erfc}\left(i \frac{\kappa}{2\sqrt{a}} \right) = \mathcal{E}(z). \tag{S7}$$

Hence, Equation S6 is given by

$$U_x(x, z) = \frac{\sqrt{\pi}}{2\sqrt{a}} \mathcal{E}(z) e^{-\frac{\kappa^2+\gamma^2}{4a}} \left(e^{\frac{\kappa\gamma}{2a}} + e^{-\frac{\kappa\gamma}{2a}} \right). \tag{S8}$$

Based on the parameters used in the simulation, where ω_0 is 10 μm, the CGB propagates at the region that satisfies $\omega_0^2 k \gg z$. Therefore, Equation S8 becomes

$$U_x(x, z) \cong \sqrt{\frac{\pi}{a}} \mathcal{E}(z) e^{-\frac{z^2\kappa^2}{k^2\omega_0^2}} e^{-\frac{iz\kappa^2}{2k}} e^{-\frac{x^2}{\omega_0^2}} e^{-\frac{ikx^2}{2z}} \cos(\kappa x). \tag{S9}$$

By substituting Equation S9 into Equation S3, the electric field is obtained by

$$\begin{aligned} E &\cong E_0 \sqrt{\frac{\pi}{a}} \frac{e^{ikz}}{i\lambda z} e^{\frac{iky^2}{2z}} U_y(y, z) \mathrm{erfc}\left(i \frac{\kappa}{2\sqrt{a}} \right) e^{-\frac{iz\kappa^2}{2k}} e^{-\frac{z^2\kappa^2}{k^2\omega_0^2}} e^{-\frac{x^2}{\omega_0^2}} \cos(\kappa x) \\ &= E_0 A'(y, z) e^{-\frac{x^2}{\omega_0^2}} \cos(\kappa x) \end{aligned} \tag{S10}$$

which shows the field profile of CGB. As the intensity of the beam is proportional to the absolute square of the electric field, the intensity of the beam along the *x*-axis is

$$\mathrm{FWHM} \approx 2 \sqrt{\frac{-\left(\frac{2}{\omega_0^2} + \kappa^2\right) + \sqrt{\left(\frac{2}{\omega_0^2} + \kappa^2\right)^2 + \frac{2}{3}\kappa^4 \ln 2}}{\frac{\kappa^4}{3}}} \cong \frac{2}{|\kappa|} \sqrt{3\left(\sqrt{1 + \frac{2}{3}\ln 2} - 1 \right)} \cong \frac{0.252\lambda_0}{\alpha n_0 d}. \tag{S11}$$

around the propagation axis where $\kappa^2 \gg \frac{2}{\omega_0^2}$ and $\kappa x \approx 1$. The propagation distance of the CGB is also calculated from Equation S10, where the intensity of the beam along the *z*-axis is

$$I \propto e^{-\frac{2z^2\kappa^2}{k^2\omega_0^2}}. \tag{S12}$$

The distance at which the intensity of the beam becomes $1/e^2$ is therefore

$$z_{\max} = \frac{k\omega_0}{\kappa} = \frac{n\omega_0}{\alpha n_0 d} \tag{S13}$$

which can also be geometrically estimated where beams from two regions overlap over this distance.

4. Analysis on cylindrical lens

Similar to Section S3, the electric field produced by the varifocal cylindrical lens is calculated for precise analysis. Due to the finite size of the metasurface, the electric field produced by the plane wave at point (x', y', 0) after passing through the metasurface is

$$E(x',y',0) = E_0 \operatorname{rect}\left(\frac{x'}{W_x}\right) \operatorname{rect}\left(\frac{y'}{W_y}\right) e^{-\frac{ik}{2f}x'^2} \tag{S14}$$

where f is the focal length of the cylindrical lens, k is the wave vector, W_x is the width of the metasurface layers along the x-axis, and W_y is the width of the overlapped region along the y-axis. Using Equation S3, the electric field at point (x, y, z) is calculated by

$$\begin{aligned} E(x,y,z) &= \frac{e^{ikz}}{i\lambda z} E_0 \int_{-W_x/2}^{W_x/2} e^{\frac{ik}{2z}(x-x')^2 - \frac{ik}{2f}x'^2}\, dx' \int_{-W_y/2}^{W_y/2} e^{\frac{ik}{2z}(y-y')^2}\, dy' \\ &= \frac{e^{ikz}}{i\lambda z} E_0 \cdot U_x(x,z) \cdot U_y(y,z) \end{aligned} \tag{S15}$$

similar to Equation S5. Here, $U_y(y, z)$ is the Fresnel diffraction caused by the slit, which does not affect the optical properties along the x-axis. Around the focus where $z = f$, the integral $U_x(x, f)$ is simplified by

$$U_x(x,f) = e^{\frac{ik}{2f}x^2} \int_{-W_x/2}^{W_x/2} e^{-\frac{ikxx'}{f}}\, dx' = e^{\frac{ik}{2f}x^2} W_x \operatorname{sinc}\left(\frac{kW_x x}{2f}\right) \tag{S16}$$

and the intensity along the x-axis at the focus is expressed as

$$I(x,f) \propto \operatorname{sinc}^2\left(\frac{\pi W_x x}{\lambda f}\right). \tag{S17}$$

Therefore, the FWHM of the cylindrical lens is calculated from Equation S17 by

$$\text{FWHM} \approx \frac{0.8858\lambda f}{W_x}. \tag{S18}$$

5. Parallelism between metasurface layers

The two metasurface layers are assumed to be parallel to each other through simulations, and the experimental setups are composed to make parallel conditions through experiments. The rotational stage (Thorlabs, RP005/M) and rotational cage mount (Thorlabs, CRM1T/M) are used for the rotational degrees of freedom, as mentioned in the Methods section of the main text. To demonstrate the parallelism between metasurface layers in the experiment setup, the distance between the two layers is measured at three points of the metasurface pattern array. Each layer is composed of an array of patterns with 6 columns and 10 rows, where the center-to-center distance is 1 mm along the *x*-axis and 500 μm along the *y*-axis. The distance between layers is measured by recording the displacement *g* required to refocus from layer 1 to layer 2 at each measurement point. Figure S4a shows the schematic of measuring parallelism, where tilt angles along the *x*-axis and *y*-axis can be calculated using

$$\tan\theta_h = (g_h - g_0)/l_1. \tag{S18}$$

$$\tan\theta_v = (g_0 - g_v)/l_2 \tag{S19}$$

where g_0, g_h, g_v denote the interlayer distance, θ_h, θ_v are the tilt angle, and l_1, l_2 are the lateral distance between two measurement points, as shown in Figure S4b and S4c. Here, l_1 is about 5 mm, and l_2 is about 4.5 mm. From the experiment, $g_h - g_0$ is measured to be about 1 μm, and $g_0 - g_v$ is measured to be about 2 μm. Therefore, the tilt angle θ_h is calculated as about 0.011°, and θ_v is approximately 0.025° from Equation S19 and S20. Based on the tilt angles and the size of the samples, it can be inferred that the bottom left side of the sample in Figure S4a has the smallest gap. The calculated tilt angles show that the layers in the experimental setup are aligned nearly parallel during the experiment.

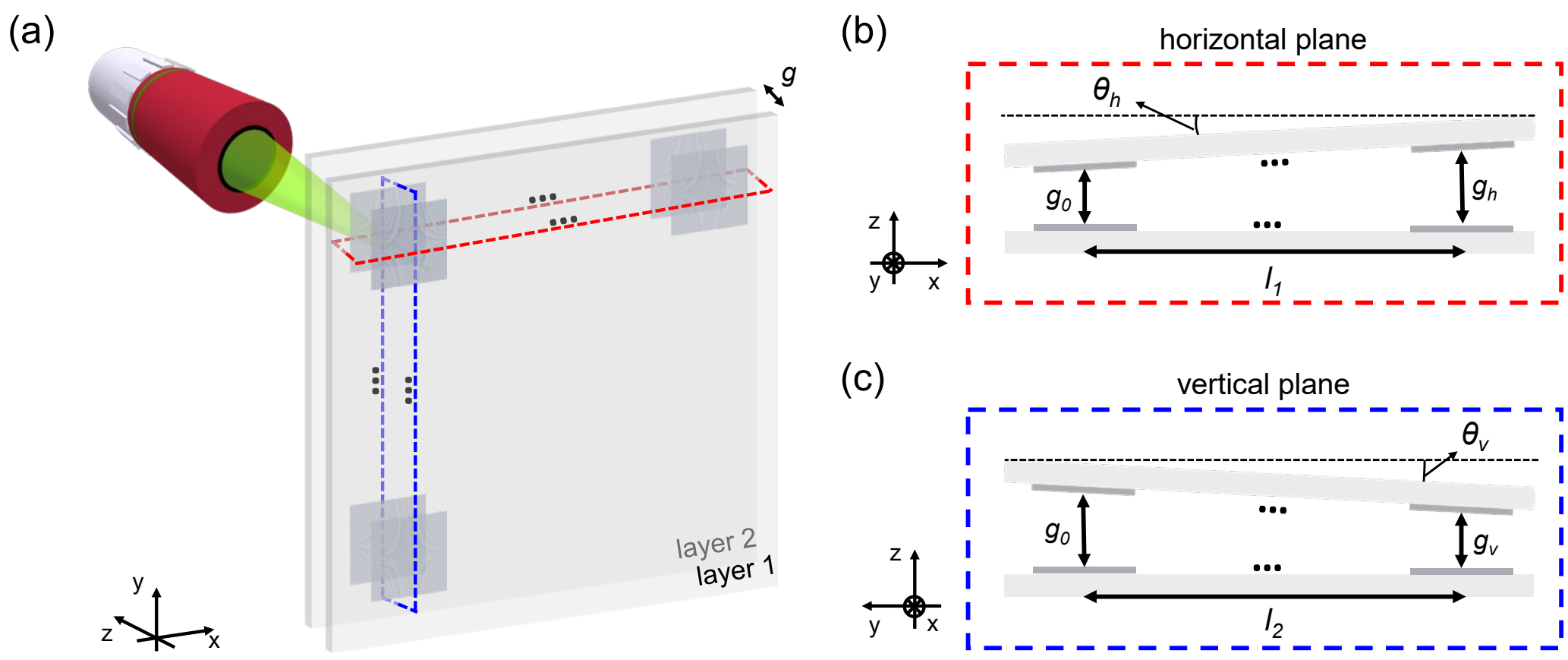

Figure S4. Measuring parallelism between metasurface layers. (a) Schematic of measuring the distance between metasurface layers. (b) Schematic of the cross-section of the *xz* plane. (c) Schematic of the cross-section of the *yz* plane.

6. Effect of interlayer distances

As briefly mentioned in the main text, a small spacing between metasurface layers is required for optimized results. However, due to the difficulties of alignment, the spacing is set much larger than the Talbot length in the experimental setup. To analyze the effect of large interlayer distances, we simulated the varifocal lens and tunable Airy beam with several distances, with a displacement of 12 μm at the varifocal lens simulations and $d = 10$ μm at the tunable Airy beam simulations. Figure S5 shows the results of the varifocal lens with different gaps between layers from 200 nm to 20 λ_0. Figure S5a shows the schematic of the simulation setup, where g is the interlayer distance from the top of the nanocylinders of layer 1 to the bottom of the nanocylinders of layer 2. Figure S5b shows the theoretical value of the focal length (black dashed line) and the simulated focal lengths (red circles). As g increases, the focal length decreases, showing that the imperfection in the experimental setup applies to the errors of experimental results in Figure 4 from the main text. Figure S5c to S5g shows the optical profiles of the varifocal lens when g is 200 nm, 3 λ_0, 5 λ_0, 10 λ_0, and 20 λ_0. On the other hand, the tunable Airy beam shows robustness to interlayer distance compared to the varifocal lens, as the Fresnel lens for the Fourier transform is only added at layer 2. Figure S6 shows the normalized intensity profiles of the tunable Airy beam with different interlayer distances. The trajectory of the Airy beam is maintained regardless of g, with some discontinuities at the first lobe and less propagation distance as g increases.

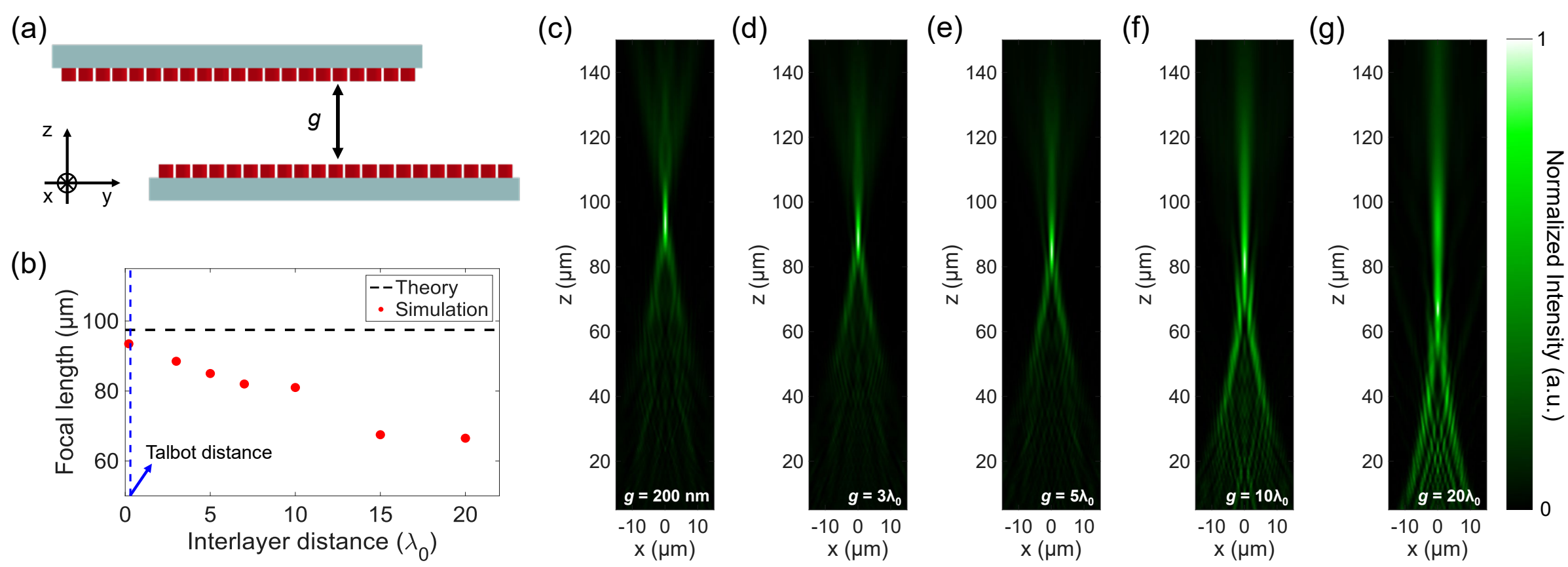

Figure S5. Simulated results of the varifocal lens by changing the interlayer distances. (a) Schematic of the simulation setup with different values of g. (b) Theoretical and simulated results of the focal lengths by changing the interlayer distances. (c) Normalized optical profile of the varifocal lens when g is 200 nm, (d) 3 λ_0, (e) 5 λ_0, (f) 10 λ_0, and (g) 20 λ_0.

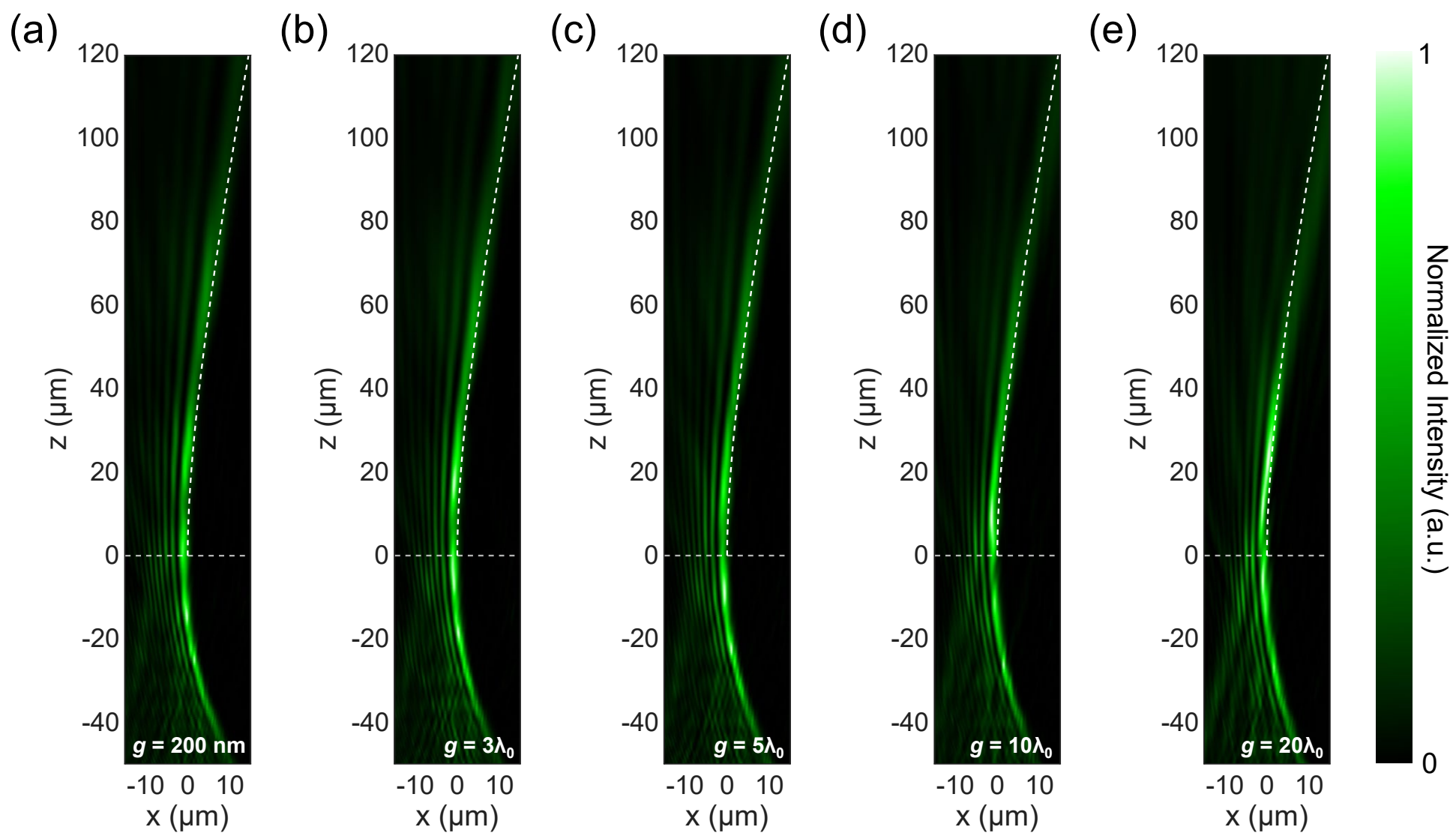

Figure S6. Simulated results of the Airy beam by changing the interlayer distances, where g is (a) 200 nm, (b) 3 λ_0, (c) 5 λ_0, (d) 10 λ_0, and (e) 20 λ_0.

7. Analysis of fabrication errors

Fabrication-induced deviations are another possible cause of the discrepancy between theoretical calculations and experimental measurements. To investigate the factors causing shorter focal length, we performed additional simulations using truncated cones instead of nanocylinders. Since the deviation in the bottom radius is relatively small, a reduced-size varifocal lens with a finer mesh is simulated to accurately capture the radius deviation. The parameter f_0 of the phase profile of the varifocal lens is 240 μm with a displacement of 8 μm. Figure S7a shows the unit cell with a bottom radius of additional deviation δ. The increase in the bottom radius results in a shorter focal length, as shown in Figure S7b, which accounts for the discrepancy from Figure 4d. Figure S7c–S7f show the intensity profiles of different δ.

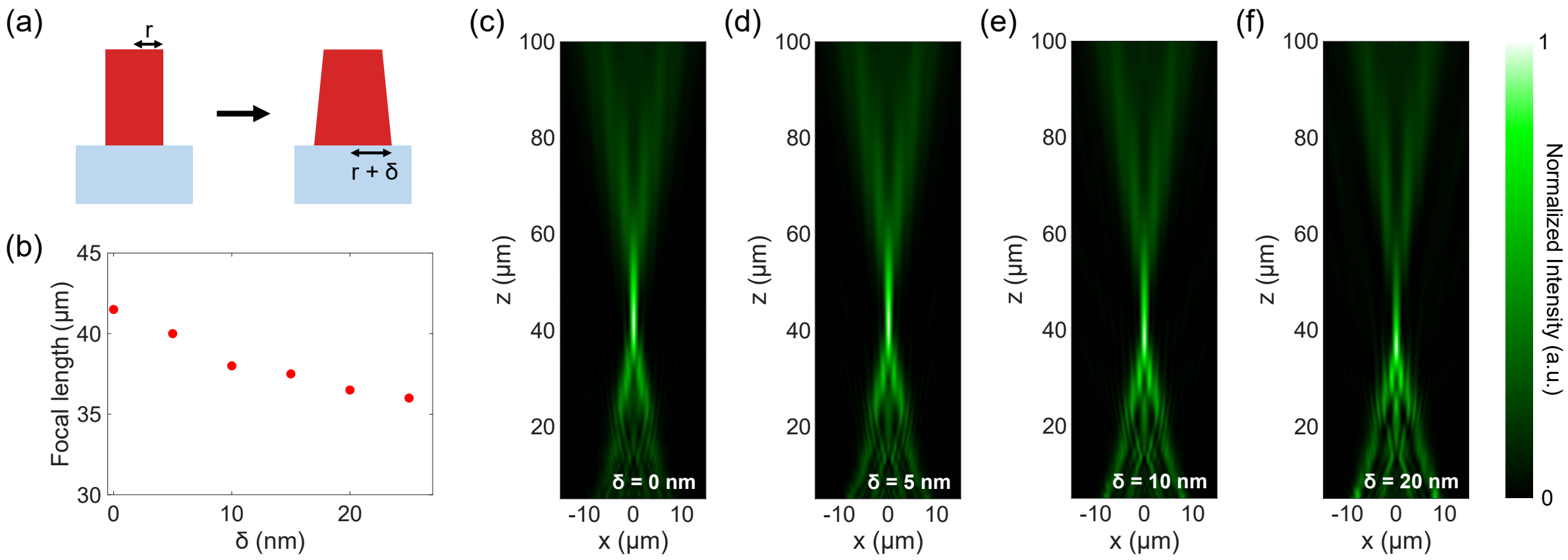

Figure S7. Simulations considering the fabrication errors. (a) Schematic of the unit cell with a bottom radius increased by δ. (b) Simulated results of focal lengths with different values of delta. (c–f) Simulated optical profiles of the varifocal lens when δ is 0 nm, 5 nm, 10 nm, and 20 nm.

8. Effects of incident light from a non-overlapping area

Throughout the simulations, the computational domain was restricted to the overlap region between the two layers, allowing the analysis of the optical field generated by the final phase profile. However, in the experiment, the incident beam also passes through the single-layer-only regions and the unpatterned substrate, which introduces unwanted contributions to the measured field. Figure S7a shows the normalized intensity profiles of the Airy beam for displacements of −10 μm, −12 μm, and −15 μm. Unlike the simulated results in Figure 3a, the first lobe of the Airy beam in Figure S7a exhibits a discontinuous trajectory along the propagation direction, which aligns with the experimental results in Figure 5. Unlike the Airy beam, the optical profiles of the varifocal lens shown in Figure S7b show no noticeable difference from the results in Figure 2d. These deviations can be mitigated by fabricating the sample larger than the spot size of the incident light.

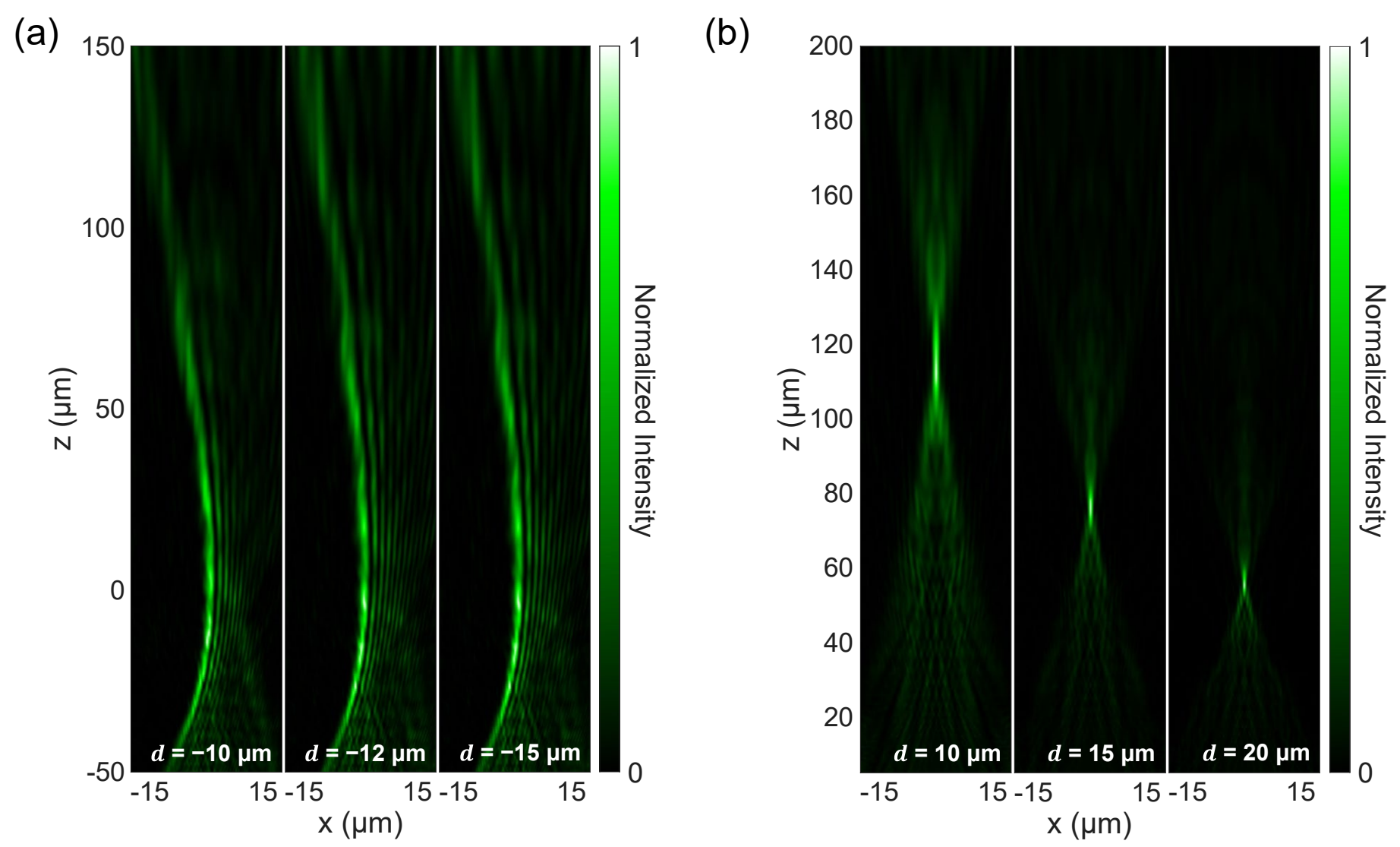

Figure S8. Simulated results including the effects of a non-overlapping area. (a) Normalized optical profiles of the Airy beam for displacements of −10 μm, −12 μm, and −15 μm. (b) Normalized optical profiles of the varifocal lens for displacements of 10 μm, 15 μm, and 20 μm.

9. Polarization independence of the metasurface

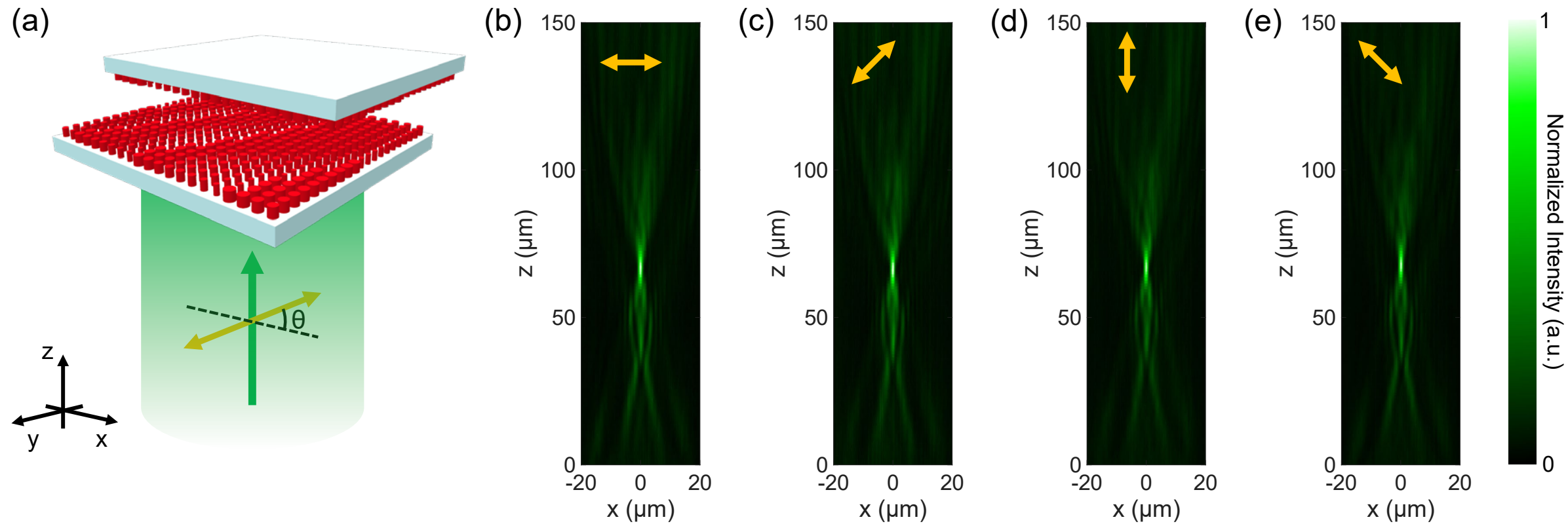

Figure S9. Experimental demonstration of polarization independence using varifocal lens samples when d = 10 μm. (a) Schematic of the setup for different incident linear polarization angles θ. (b–e) Measured field images of the varifocal lens when θ is 0°, 45°, 90°, and 135°.

References

1. J. Lin, J. Dellinger, P. Genevet, B. Cluzel, F. de Fornel, and F. Capasso, "Cosine-Gauss plasmon beam: a localized long-range nondiffracting surface wave," *Physical Review Letters* 109, no. 9 (2012): 093904. https://doi.org/10.1103/PhysRevLett.109.093904
